# A Rare Case of Cemento-Ossifying Fibroma of the Maxilla

**DOI:** 10.5334/jbsr.2863

**Published:** 2022-09-13

**Authors:** Elyn Van Snick, Bjorn Valgaeren, Jan Hendrickx

**Affiliations:** 1 Vrije Universiteit Brussel, BE; 2 AZ Damiaan Oostende, BE

**Keywords:** cemento-ossifying fibroma, bone lesion, osteogenic tumours, cone-beam computed tomography

## Abstract

**Teaching Point:** Cemento-ossifying fibromas are rare, benign tumours that are mostly found in the tooth-bearing areas of the mandible or maxilla and can be seen on cone-beam computed tomography.

## Case History

A 20-year-old woman presented at the maxillofacial surgeon because of a painless submucosal swelling under the right upper lip that had been present for over one year.

**Figure 1.**
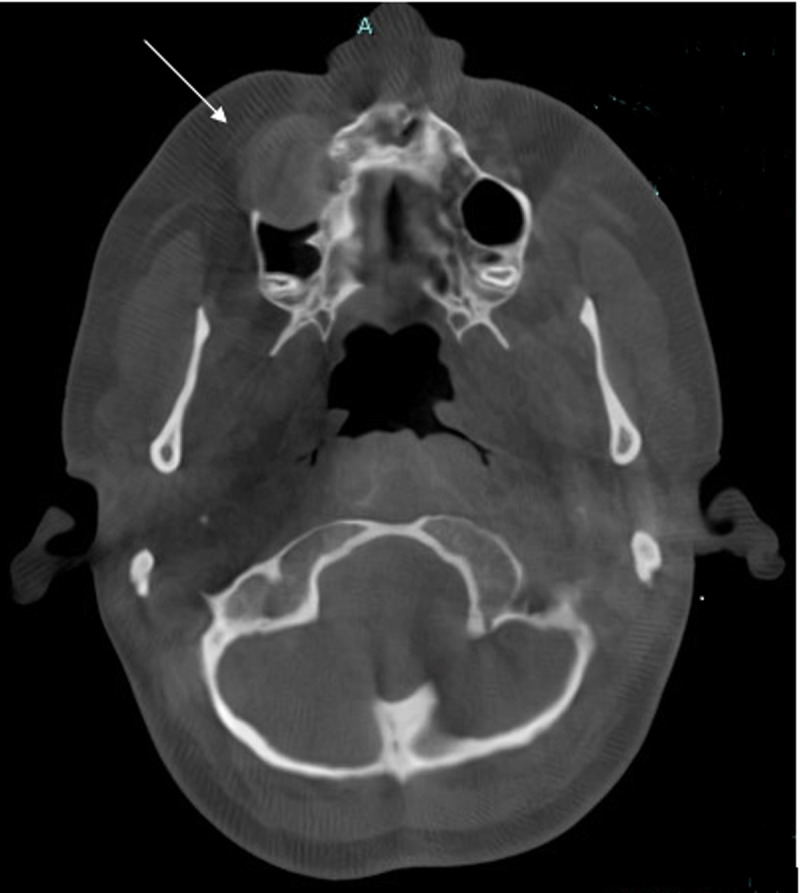


**Figure 2:**
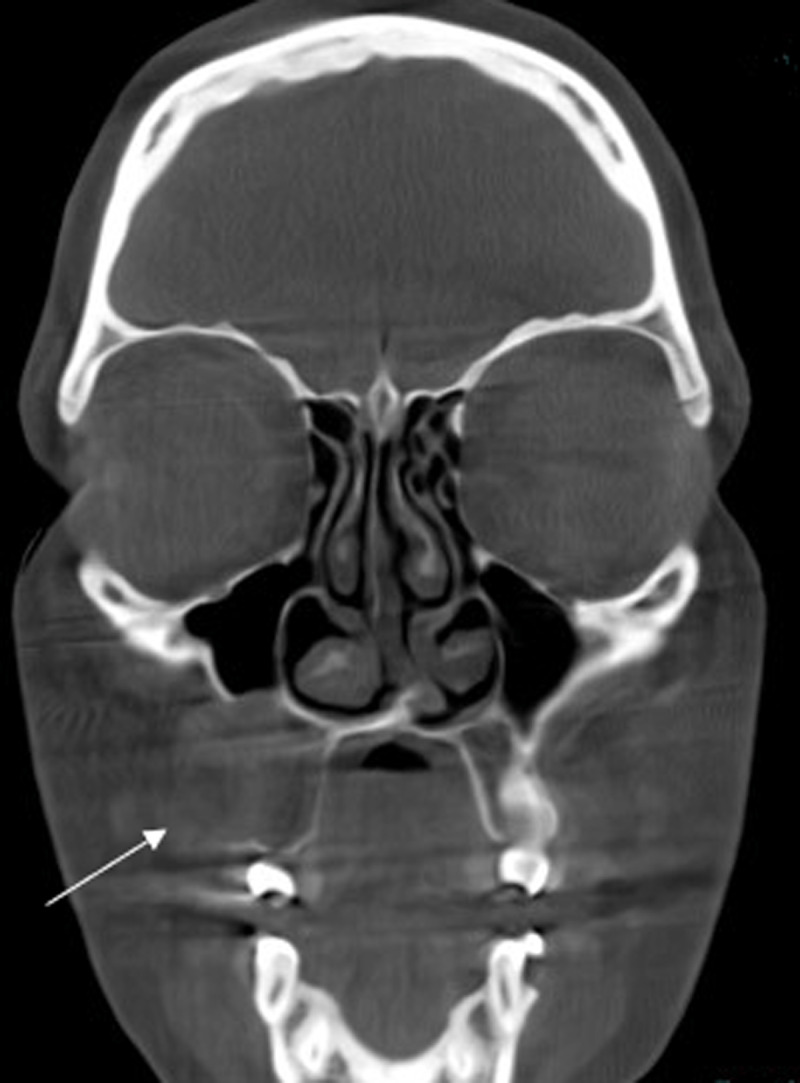


Cone-beam computed tomography (CBCT) scan showed a sharply demarcated hypodense nodule (2.1 cm) centred in the space between the roots of tooth element 14 and 15 (Figures 1 and 2).

**Figure 3:**
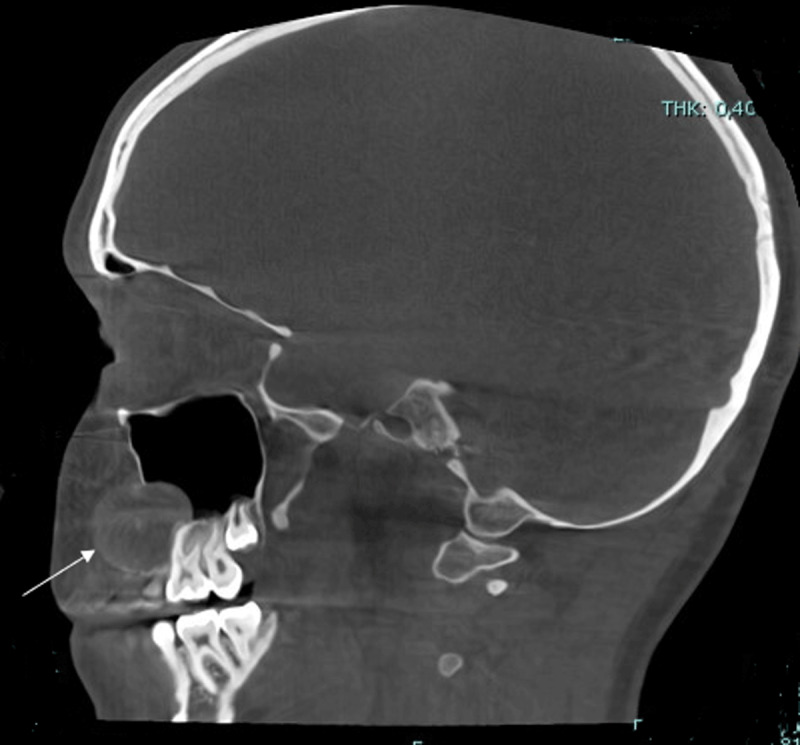


The lesion had an expansive character with loss of cortical delineation of the anterior wall of the right maxillary sinus (Figure 3).

The lesion was surgically removed and was histologically proven to be a cemento-ossifying fibroma.

## Comment

Cemento-ossifying fibromas are rare, benign tumours that are mostly found in the tooth-bearing areas of the mandible or maxilla. The 2005 World Health Organisation (WHO) classification considers them as osteogenic tumours. They consist of fibrous tissue and various amounts of calcified tissue and cementum. Their origin is thought to be the periodontal ligament, which explains their usual close relationship to the teeth [1].

Cemento-ossifying fibromas mostly occur during the third and fourth decades of life and have a female predilection. Patients often present with a slow growing solid mass, that may displace the teeth [1].

On plain radiographs, cemento-ossifying fibromas present as well-circumscribed lucent lesions. On computed tomography (CT) scan they show soft tissue attenuation and they usually enhance after administration of intravenous iodinated contrast. Calcifications develop as the lesions mature [1]. CBCT can provide a detailed depiction of the location and expansion of the lesion, as well as its margins in relation to the surrounding osseous structures and teeth. This may aid both in diagnosis as in the planning of treatment. On magnetic resonance imaging (MRI) the lesions are T1 hypointense or isointense and T2 hypointense. They may enhance after intravenous administration of gadolinium contrast [1].

Treatment consists of surgical excision. Postoperative recurrence is considered rare [1].

The differential diagnosis, depending on the degree of calcification includes (but is not limited to): juvenile ossifying fibroma, odontoma, odontogenic cyst, cementoblastoma, fibrous dysplasia, osteosarcoma, osteochondroma, and chondrosarcoma [1].
